# Association of Atopic Dermatitis With Sleep Quality in Children

**DOI:** 10.1001/jamapediatrics.2019.0025

**Published:** 2019-03-04

**Authors:** Faustine D. Ramirez, Shelley Chen, Sinéad M. Langan, Aric A. Prather, Charles E. McCulloch, Sharon A. Kidd, Michael D. Cabana, Mary-Margaret Chren, Katrina Abuabara

**Affiliations:** 1 Department of Dermatology Program for Clinical Research, University of California, San Francisco, San Francisco; 2 Faculty of Epidemiology and Population Health, London School of Hygiene and Tropical Medicine, London, United Kingdom; 3 Department of Psychiatry, University of California, San Francisco, San Francisco; 4 Department of Epidemiology & Biostatistics, University of California, San Francisco, San Francisco; 5 Department of Pediatrics, University of California, San Francisco, San Francisco; 6 Philip R. Lee Institute for Health Policy Studies, University of California, San Francisco, San Francisco; 7 Department of Dermatology, Vanderbilt University Medical Center, Nashville, Tennessee

## Abstract

**Importance:**

Pruritus, a hallmark of atopic dermatitis (AD), is thought to disrupt sleep, yet little is known about how variations in disease activity and severity of this common childhood condition may be associated with sleep patterns over time.

**Objective:**

To determine whether children with active AD have impaired sleep duration and quality at multiple time points throughout childhood and whether disease severity affects sleep outcomes.

**Design, Setting, and Participants:**

This longitudinal cohort study used data of children enrolled in the Avon Longitudinal Study of Parents and Children, a population-based birth cohort in Avon, United Kingdom. Participants were children (N = 13 988) alive at 1 year and followed up with repeated measures of self-reported AD and sleep through 16 years of age. This study was based on data collected from 1990 to 2008. Data analysis was performed from September 2017 to September 2018.

**Main Outcomes and Measures:**

Standardized measure of sleep duration and composite measure of sleep quality, including nighttime awakenings, early morning awakenings, difficulty falling asleep, and nightmares, were repeated at multiple time points between 2 and 16 years of age.

**Results:**

The study sample comprised 13 988 children (7220 male [51.6%]) followed up for a median (interquartile range [IQR]) duration of 11 (5-14) years. Of this total, 4938 children (35.3%) met the definition of having atopic dermatitis between 2 and 16 years of age. Total sleep duration was similar between children with active AD and without AD at all ages, and the average estimated difference across childhood was a clinically negligible difference of 2 minutes less per day for children with AD (95% CI, −4 to 0 minutes). In contrast, children with active AD were more likely to report worse sleep quality at all time points, with a nearly 50% higher odds of experiencing more sleep-quality disturbances (adjusted odds ratio [aOR], 1.48; 95% CI, 1.33 to 1.66). Children with more severe active disease (quite bad or very bad AD: aOR, 1.68; 95% CI, 1.42 to 1.98) and with comorbid asthma or allergic rhinitis (aOR, 1.79; 95% CI, 1.54 to 2.09) had worse sleep quality. However, even children with mild AD (OR, 1.40; 95% CI, 1.27 to 1.54) or inactive AD (OR, 1.41; 95% CI, 1.28 to 1.55) had statistically significantly increased odds of impaired sleep quality.

**Conclusions and Relevance:**

Atopic dermatitis appeared to be associated with impaired sleep quality throughout childhood; thus, it is suggested that clinicians should consider sleep quality among all children with AD, especially those with comorbid asthma or allergic rhinitis and severe disease, and that interventions to improve sleep quality are needed.

## Key Points

**Question:**

Do children with atopic dermatitis experience impaired sleep duration and sleep quality throughout childhood, and do disease severity and activity affect their sleep?

**Findings:**

In this longitudinal cohort study of 13 988 children, atopic dermatitis was statistically significantly associated with impaired sleep quality but not sleep duration throughout childhood. Sleep impairment was more common among children with more severe disease and with comorbid asthma or allergic rhinitis, and the risk remained elevated even among children with mild and inactive atopic dermatitis.

**Meaning:**

These findings suggest that clinicians should consider sleep quality among all children with atopic dermatitis, especially those with comorbid asthma or allergic rhinitis and severe disease; it appears interventions to improve sleep quality are needed for this population.

## Teaser

This cohort study examines the prevalence and severity of atopic dermatitis as well as sleep duration and quality at multiple periods among young children enrolled in the Avon Longitudinal Study of Parents and Children in the United Kingdom.

## Introduction

Atopic dermatitis ranks among the largest components of the nonfatal disease burden worldwide.^1^ Sleep disturbances have been identified as central to quality-of-life decrements in atopic dermatitis,^2,3^ but little is known about their association with sleep in the general population. Pruritus, a hallmark of atopic dermatitis, is often worst at night, resulting in scratching that may interfere with the process of falling asleep and cause disruptions in ongoing sleep.^4,5^ Small polysomnography and actigraphy studies among clinic-based populations have found that children with atopic dermatitis are more restless in their sleep, awaken more often, and spend more time awake after the onset of sleep.^6,7,8,9,10^ Adequate sleep is critical to well-being and health; in children, acute and chronic sleep disturbances have been associated with a wide range of cognitive, mood, and behavioral impairments and have been linked to poor educational performance.^11,12,13^

Atopic dermatitis has a chronic relapsing and remitting course, and it is unknown how variations in disease activity and severity affect sleep at different periods throughout childhood. Longitudinal studies can help characterize and quantify the burden of atopic dermatitis–associated sleep loss during these critical developmental periods. We aimed to determine whether children with active atopic dermatitis have impaired sleep duration and quality throughout childhood and whether the severity of atopic dermatitis affects sleep outcomes in a population-based birth cohort.

## Methods

We performed a longitudinal cohort study using data collected from 1990 to 2008 from the Avon Longitudinal Study of Parents and Children (ALSPAC). Participants in ALSPAC provided written informed consent, and ethical approval was obtained from the ALSPAC Ethics and Law Committee and the Local Research Ethics Committees. The present study was considered exempt from review by the University of California, San Francisco Institutional Review Board because all of the data obtained by investigators were fully deidentified. Data analysis was performed from September 2017 to September 2018.

### Participants

Pregnant women residing in Avon, United Kingdom, were recruited between 1990 and 1992 and followed up in the ensuing 2 decades with standardized questionnaires and clinical assessment visits, as described in detail elsewhere.^14,15^ The ALSPAC study enrolled a total of 14 541 pregnancies, which resulted in 14 062 live births, of which 13 988 were alive at 1 year of age. The current study sample is limited to children alive at 1 year of age and includes assessments through age 16 (n = 11 620; 83% of those alive at 1 year of age). The ALSPAC website contains details of all of the data available through a fully searchable data dictionary and variable search tool.^16^

### Exposure

The primary exposure was atopic dermatitis annual period prevalence, measured by a standardized question about flexural (joints and creases) dermatitis answered by the mother or the child (latest time point only) at 12 time points between age 6 months and 16 years: Has your child had an itchy, dry skin rash in the joints and creases of his body (eg, behind the knees, elbows, under the arms) in the past year? This question is comparable to that used in the large International Study of Asthma and Allergies in Childhood (ISAAC).^17^ Individuals were considered to have active atopic dermatitis if they had at least 2 reports of flexural dermatitis, up to and including the time point being considered.^18,19,20^ On the first report of flexural dermatitis, individuals were categorized as being indeterminate for atopic dermatitis and not included in the control group for that time point. Disease severity was assessed at each time point by a question that asked mothers to categorize their child’s disease over the past year as no problem, mild, quite bad, or very bad. Finally, children were classified as having inactive atopic dermatitis if they met the definition of *active* atopic dermatitis previously but responded negatively at the time point being considered.

### Sleep Outcomes

Sleep duration was assessed by standardized questionnaires at 8 time points (30, 42, 57, 69, 81, 115, 140, and 186 months) between ages 2 and 16 years. Nighttime sleep duration was calculated on the basis of maternal or self-report (16 years only) of the time the child usually went to sleep and usually woke up in the morning. Mothers were also asked about daytime sleep duration at 5 time points between ages 2 and 7 years. Total sleep duration was calculated by adding nighttime and daytime duration up until age 7 years, and it was equal to nighttime sleep duration alone after age 7 years. Both nighttime and total sleep duration were found to be approximately normally distributed.

Sleep quality was assessed using 4 standardized questions at 6 time points (30, 42, 57, 69, 81, and 115 months) between ages 2 and 10 years. Mothers were asked about nighttime awakenings (≥1 per night) and whether the child regularly experienced early morning awakenings, difficulty falling asleep, and nightmares over the past year. Responses were analyzed individually and combined into a composite sleep-quality outcome scored from 0 to 4, assigning 1 point for each item.

### Additional Covariates

**Figure 1.**
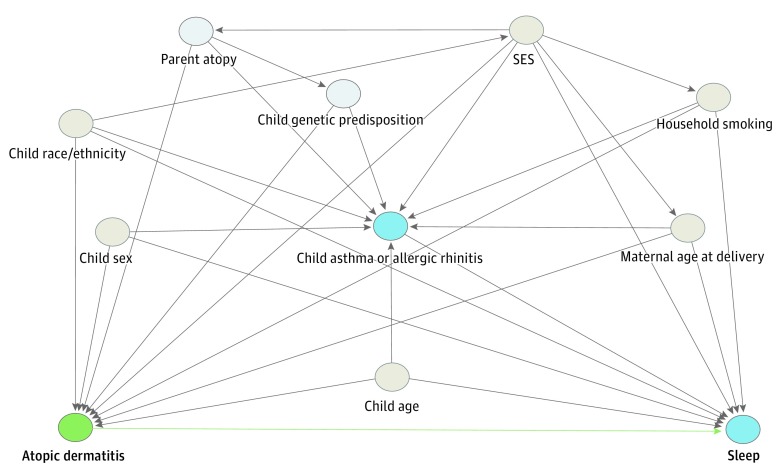
Directed Acyclic Graph A directed acyclic graph represents associations between covariates and primary exposure and outcome. Gray circles represent ancestors of the exposure and outcome (ie, confounders), blue circles represent ancestors of the outcome (ie, causal determinants of the outcome), and light blue circles represent unobserved (ie, latent) variables. Green lines represent causal paths, and gray lines represent biasing paths. The minimally sufficient adjustment set represents covariates such that the adjustment for this set of variables will minimize confounding bias when estimating the association between the exposure and the outcome. The minimally sufficient adjustment set was determined using the DAGitty software.^21^ Child comorbid asthma or allergic rhinitis was considered to be a collider, which was appropriately accounted for by adjusting for additional variables contained on the backdoor paths shared by this collider. The final minimally sufficient adjustment set comprised child sex, age, race/ethnicity, and comorbid asthma or allergic rhinitis; maternal age at delivery; socioeconomic status (SES); and household smoking exposure.

Potential confounders and effect modifiers were identified from the literature and incorporated into a directed acyclic graph that was used to guide the modeling strategy (Figure 1).^22,23,24,25,26,27^ These covariates included child and mother demographic characteristics (child sex, age, and race/ethnicity as well as maternal age at delivery), indicators of socioeconomic status, household smoking exposure, and comorbid asthma or allergic rhinitis. Socioeconomic status was measured using prenatal questionnaires collected from parents at study enrollment, including the highest educational qualification of the mother; social class based on occupation (highest of either parent); household crowding index (number of people living in the household divided by the number of rooms in the house); and a financial difficulties score assessing the mother’s self-reported difficulty to afford food, clothing, heating, rent or mortgage, and items necessary to care for her baby.

Time-varying covariates included comorbid asthma or allergic rhinitis and household smoking exposure. A child was determined to have comorbid asthma or allergic rhinitis at each time point examined if the mother reported asthma and/or allergic rhinitis symptoms at that time point, based on standardized questions similar to those used in the ISAAC study.^17^ Finally, models also included a measure of household smoking exposure, assessed by a maternal questionnaire about the number of smokers living in the household at multiple time points throughout childhood.

### Missing Data

As has been described in detail elsewhere, the ALSPAC cohort has both intermittent missing data and attrition (ie, loss to follow-up).^14^ For example, the mean response rate to 12 surveys during the adolescence phase was 48%, but 75% of individuals responded at least once during adolescence. Multiple imputation was performed to impute missing exposure, outcome, and covariate data. Iterative chained equations were used, as most variables in the models did not follow a normal distribution. Fifty imputed data sets were generated and used to repeat primary analyses.

### Statistical Analysis

Cross-sectional regression analyses were performed to compare sleep outcomes between children with and children without atopic dermatitis at each time point (linear models for sleep duration, logistic models for binary sleep-quality outcomes, and ordered logistic regression models for the composite sleep-quality measure). Longitudinal analyses with repeated measures of the exposure, outcome, and time-varying covariates (asthma or allergic rhinitis and household smoking exposure) were then conducted using mixed-effects models with random slopes and intercepts for each individual. For all longitudinal analyses, table legends include the total number of individuals included in each model and the mean number of observations (ie, number of time points used) per individual. The minimally sufficient adjustment set was determined using a directed acyclic graph (Figure 1). We tested for interactions between atopic dermatitis and comorbid asthma or allergic rhinitis, child’s age, child sex, and maternal educational level. Of these variables, only the interaction with comorbid asthma or allergic rhinitis was found to be statistically significant for sleep-quality outcomes (*P* < .05); thus, these results are presented stratified by asthma or allergic rhinitis status. All statistical analyses were performed using Stata, version 14.2 (StataCorp Inc).

## Results

The study sample was composed of 13 988 children (7220 male [51.7%]) followed up for a median (interquartile range [IQR]) duration of 11 (5-14) years. Overall, 4938 children (35.3%) met the definition of atopic dermatitis between 2 and 16 years of age. Children with atopic dermatitis were more likely to be female, have comorbid asthma or allergic rhinitis, have a family history of atopic conditions, be from a family of a high social class, and have a mother with a high level of education (Table 1). The annual period prevalence of active atopic dermatitis ranged from 13% to 21% from age 2 to 16 years, and 22% to 40% of individuals with atopic dermatitis reported quite bad to very bad disease at any given time point (eTable 1 in the Supplement).

**Table 1.** Cohort Characteristics

Variable	Total, No.	No. (%)	Without Atopic Dermatitis (n = 5555)	With Atopic Dermatitis (n = 4938)^a^	*P* Value^b^
Child, No. (%)					
Male sex	13 978	7220 (51.7)	3008 (54.2)	2377 (48.1)	<.001
White race/ethnicity	12 077	11 468 (95.0)	4755 (95.5)	4490 (95.7)	.65
Asthma ever^c^	12 612	3237 (25.7)	1058 (19.1)	1789 (36.2)	<.001
Allergic rhinitis ever^c^	10 156	1375 (13.5)	339 (7.8)	913 (20.3)	<.001
Asthma or allergic rhinitis ever^c^	12 620	3919 (31.1)	1272 (22.9)	2188 (44.3)	<.001
Asthma and allergic rhinitis ever^c^	12 620	693 (5.5)	125 (2.3)	514 (10.4)	<.001
Maternal age at delivery, No. (%), y					<.001
≤20	13 972	1004 (7.2)	375 (6.8)	212 (4.3)	
21-34	13 972	11 585 (82.9)	4589 (82.6)	4196 (85.0)	
≥35	13 972	1383 (9.9)	588 (10.6)	530 (10.7)	
History of atopic condition, No. (%)^d^					
Maternal	12 454	5659 (45.4)	2154 (42.0)	2402 (50.3)	<.001
Paternal	8545	3418 (40.0)	1307 (36.8)	1543 (45.2)	<.001
Household smoking exposure, No. (%)^e^	10 188	3683 (36.2)	1643 (37.7)	1476 (33.5)	<.001
Maternal highest educational level, No. (%)^f^					<.001
CSE/none	12 412	2504 (20.2)	1101 (21.5)	726 (15.2)	
Vocational	12 412	1224 (9.9)	510 (9.9)	446 (9.3)	
O level	12 412	4294 (34.6)	1833 (35.7)	1656 (34.7)	
A level	12 412	2791 (22.5)	1130 (22.0)	1182 (24.8)	
Degree	12 412	1599 (12.9)	559 (10.9)	762 (16.0)	
Social class, No. (%)^g^					<.001
Unskilled	12 254	227 (1.9)	86 (1.7)	54 (1.2)	
Partly skilled	12 254	920 (7.5)	375 (7.5)	285 (6.2)	
Skilled manual	12 254	1666 (13.6)	712 (14.2)	516 (11.2)	
Skilled nonmanual	12 254	3780 (30.8)	1582 (31.6)	1390 (30.2)	
Managerial and technical	12 254	4566 (37.3)	1862 (37.2)	1846 (40.1)	
Professional	12 254	1095 (8.9)	391 (7.8)	508 (11.1)	
Financial difficulties quartile, No. (%)					.08
Lowest	12 083	4337 (35.9)	1798 (35.9)	1781 (38.4)	
Mild	12 083	3006 (24.9)	1254 (25.1)	1142 (24.6)	
Moderate	12 083	2324 (19.2)	970 (19.4)	862 (18.6)	
Highest	12 083	2416 (20.0)	979 (19.6)	851 (18.4)	
Crowding index, No. (%)^h^					<.001
<0.5	12 799	5329 (41.6)	2058 (39.9)	2231 (46.9)	
>0.5-0.75	12 799	4013 (31.3)	1623 (31.5)	1509 (31.7)	
>0.75-1	12 799	2579 (20.2)	1106 (21.5)	810 (17.0)	
>1	12 799	878 (6.9)	368 (7.1)	207 (4.4)	

Abbreviation: CSE, Certificate of Secondary Education.

^a^ Children who met the definition of atopic dermatitis by age 16 years (ie, had at least 2 reports of flexural dermatitis). There were 3505 individuals with only 1 report of flexural dermatitis.

^b^ χ^2^ Test comparing children who never reported atopic dermatitis with children who ever reported atopic dermatitis through age 16 years.

^c^ At least 2 reports of symptoms throughout childhood.

^d^ Including atopic dermatitis, asthma, or allergic rhinitis.

^e^ At 1.75 years of age.

^f^ UK educational levels: CSE, certificate after passing national school examinations at 16 years of age; vocational; O level, qualification after passing national school examinations at 16 years of age; higher than CSE; A level, qualification after passing national school examinations at 18 years of age; degree, university degree, or higher.

^g^ Highest of either parent, based on occupation.

^h^ The number of persons living in the household divided by the number of rooms in that household.

### Sleep Duration

The mean (SD) nighttime sleep duration ranged from 11.2 (1.0) hours at 2 years of age to 8.7 (0.9) hours at 16 years of age. The median (IQR) daytime sleep duration was 30 (0-90) minutes at 2 years of age and 0 minutes thereafter. Overall, throughout childhood, nighttime sleep duration was similar between children with active atopic dermatitis and those without atopic dermatitis (Table 2). In adjusted models, the estimated difference was 0 minutes (95% CI, −2 to 2), and we did not find any statistically significant differences or gradient by atopic dermatitis severity levels. For total sleep duration (including daytime naps though age 7 years), we found a statistically significant but clinically negligible difference: individuals with active atopic dermatitis were estimated to sleep a mean 2 minutes less per day throughout childhood (95% CI, −4 to 0), and this association was similar across all disease severity levels (Table 2).

**Table 2.** Estimated Differences in Sleep Duration According to Atopic Dermatitis Disease Activity and Severity^a^

Disease Activity and Severity	Estimated Difference in Sleep Duration (95% CI), min	
	Unadjusted	Adjusted
Nighttime sleep duration^b^^,^^c^		
Never reported atopic dermatitis	1 [Reference]	1 [Reference]
Overall active atopic dermatitis	−8 (−10 to −6)	0 (−2 to 2)
No problem	−9 (−13 to −6)	1 (−2 to 4)
Mild	−11 (−13 to −9)	1 (−1 to 3)
Quite bad	−7 (−10 to −5)	0 (−2 to 2)
Very bad	−5 (−10 to 0)	−1 (−6 to 3)
Inactive atopic dermatitis	−33 (−35 to −32)	2 (1 to 4)
Total sleep duration^d^^,^^e^		
Never reported atopic dermatitis	1 [Reference]	1 [Reference]
Overall active atopic dermatitis	−10 (−13 to −8)	−2 (−4 to 0)
No problem	−15 (−19 to −10)	−2 (−5 to 1)
Mild	−17 (−19 to −14)	−2 (−4 to −1)
Quite bad	−8 (−11 to −5)	−1 (−4 to 1)
Very bad	−3 (−8 to 2)	−3 (−7 to 1)
Inactive atopic dermatitis	−43 (−45 to −42)	−1 (−3 to 0)

^a^ Results from unadjusted and adjusted multivariable mixed-effects linear regression models examining the association between atopic dermatitis and nighttime and total sleep duration at 8 time points (30, 42, 57, 69, 81, 115, 140, and 186 months) between ages 2 and 16 years. The multivariable model adjusted for potential confounders, including child’s sex, age, race/ethnicity, and comorbid asthma or allergic rhinitis; household smoking exposure; maternal educational level, social class, and age at delivery; crowding index; and financial difficulties score.

^b^ Unadjusted model with 11 549 individuals; mean (range) of 5.3 (1-8) observations per individual.

^c^ Adjusted model with 9109 individuals; mean (range) of 5.0 (1-7) observations per individual.

^d^ Unadjusted model with 11 531 individuals; mean (range) of 5.3 (1-8) observations per individual.

^e^ Adjusted model with 9101 individuals; mean (range) of 5.0 (1-7) observations per individual.

### Sleep Quality

**Figure 2.**
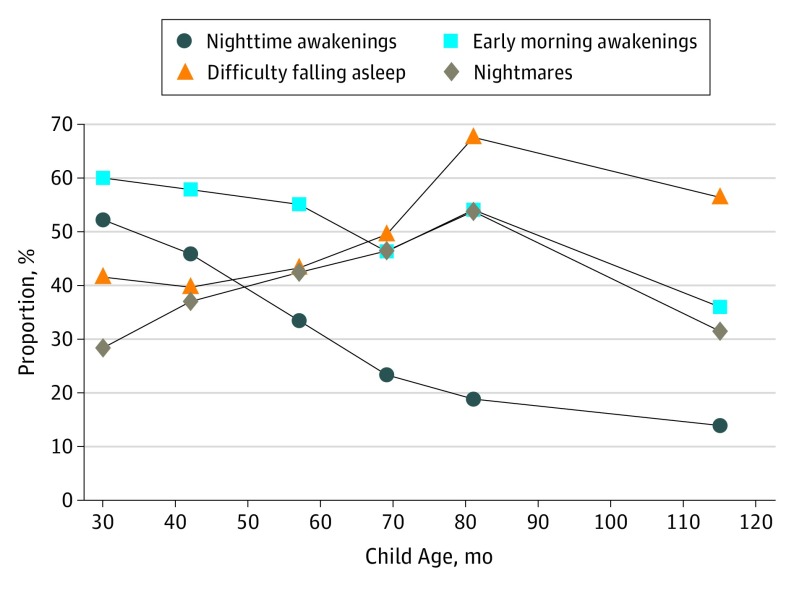
Proportion of Children With Active Atopic Dermatitis Experiencing Sleep-Quality Disturbances by Child Age Proportion of children with active atopic dermatitis reporting each of the 4 sleep-quality disturbances based on cross-sectional data at different child ages.

At any point between 2 and 10 years of age, the mean number of sleep disturbances ranged from 1.3 to 1.8, and 72% to 87% of the population experienced 1 or more sleep-quality disturbances. Overall, 5075 (50.0%) of 10 159 children reported regularly waking at least once in the night at age 2 years, which decreased to 1001 (13.5%) of 7435 children by age 10 years. A large proportion of all children reported regularly waking early in the morning (36.3% [2806 of 7739] to 58.2% [5930 of 10 195]), regularly having difficulty falling asleep (37.1% [3729 of 10 047] to 63.0% [5312 of 8434]), and regularly experiencing nightmares (26.2% [2667 of 10 166] to 49.5% [4138 of 8364]) at any given time point. The proportion of children with active atopic dermatitis experiencing all 4 sleep-quality disturbances according to child’s age is shown in Figure 2.

In cross-sectional analyses, children with active atopic dermatitis were more likely to report worse sleep-quality outcomes at all ages (eTable 2 in the Supplement). We found evidence for statistically significant interaction between atopic dermatitis and comorbid asthma or allergic rhinitis for the occurrence of impaired sleep-quality outcomes (overall test for interaction *P* = .04; Table 3). Children with only active atopic dermatitis had nearly 50% higher odds of reporting more sleep-quality disturbances throughout childhood (adjusted odds ratio [aOR], 1.48; 95% CI, 1.33-1.66), compared with those who never reported atopic dermatitis. Children with both active atopic dermatitis and either asthma or allergic rhinitis had nearly 80% increased odds of reporting more sleep-quality disturbances throughout childhood (aOR, 1.79; 95% CI, 1.54-2.09). In comparison, those with only asthma or allergic rhinitis and no atopic dermatitis had about 40% greater odds of reporting more sleep-quality disturbances throughout childhood (aOR, 1.42; 95% CI, 1.26-1.60).

**Table 3.** Adjusted Participant-Specific Odds of Reporting More Sleep-Quality Disturbances According to Atopic Dermatitis Disease Activity and Severity^a^^,^^b^

Disease Activity and Severity	Odds Ratio (95% CI)	
	Without Asthma or Allergic Rhinitis	With Asthma or Allergic Rhinitis
Never reported atopic dermatitis	1 [Reference]	1 [Reference]
Overall active atopic dermatitis	1.48 (1.33-1.66)	1.79 (1.54-2.09)
No problem	1.24 (1.04-1.48)	1.52 (1.07-2.16)
Mild	1.40 (1.27-1.54)	1.47 (1.26-1.71)
Quite bad	1.51 (1.32-1.73)	2.03 (1.66-2.48)
Very bad	1.85 (1.41-2.45)	2.28 (1.65-3.15)
Inactive atopic dermatitis	1.41 (1.28-1.55)	1.52 (1.31-1.77)

^a^ Results from an adjusted multivariable mixed-effects ordinal logistic regression model examining the association between atopic dermatitis and a composite measure of sleep quality (including nighttime awakenings, early morning awakenings, difficulty going to sleep, and nightmares) at 6 time points (30, 42, 57, 69, 81, and 115 months) between ages 2 and 10 years. The model adjusted for potential confounders, including child’s sex, age, race/ethnicity, and comorbid asthma or allergic rhinitis; household smoking exposure; maternal educational level, social class, and age at delivery; crowding index; and financial difficulties score as well as an interaction term between atopic dermatitis and comorbid asthma or allergic rhinitis. Model with 9112 individuals; mean (range) of 4.5 (1-6) observations per individual.

^b^ Overall test for interaction: *P* = .04.

More severe disease was associated with worse sleep-quality outcomes, among children with and without comorbid asthma or allergic rhinitis (Table 3). For those with quite bad or very bad active disease, children with only active atopic dermatitis had nearly 1.7 times the odds of reporting more sleep-quality disturbances throughout childhood (aOR, 1.68; 95% CI, 1.42-1.98), and those with both active atopic dermatitis and comorbid asthma or allergic rhinitis had 2.15 times the odds of reporting more sleep-quality disturbances throughout childhood (aOR, 2.15; 95% CI, 1.75-2.64), compared with those who never reported atopic dermatitis. Children with inactive disease had similar odds of reporting more sleep-quality disturbances (OR, 1.41; 95% CI, 1.28-1.55) as those with active mild disease (OR, 1.40; 95% CI, 1.27-1.54), and both were statistically significantly higher than the reference group (Table 3). These results were largely consistent across individual sleep-quality outcomes (eTables 3-6 in the Supplement).

### Multiple Imputation Results

Primary analyses yielded results that were largely consistent with those estimated from the imputed data (eTables 3-8 in the Supplement). For sleep quality, estimates using the imputed data were slightly attenuated toward the null; however, results remained qualitatively similar and statistically significant. For sleep duration, the results were nearly identical.

## Discussion

Among the 13 988 children from the ALSPAC cohort followed up from birth through adolescence, we found similar sleep duration between children with active atopic dermatitis and those without. In contrast, children with active atopic dermatitis experienced worse sleep quality throughout childhood. This association was largest among children with more severe disease and among children with comorbid asthma or allergic rhinitis, but it remained statistically significant even for those with inactive and mild disease.

These findings are consistent with those of small cross-sectional studies of clinic populations that used objective measures of sleep, including actigraphy and polysomnography. In those studies, despite increases in sleep fragmentation and reductions in sleep efficiency, overall sleep duration was similar between children with and without atopic dermatitis.^6,7,8,9,10^ In contrast, time spent awake after sleep onset is consistently greater among children with atopic dermatitis, ranging from approximately 45 to 100 minutes.^6,7,8,9^ In addition to increased nighttime awakenings and difficulty falling asleep, we found that children with active atopic dermatitis were more likely to report nightmares and early morning awakenings, which has not been previously studied.^10^

A strength of this longitudinal study was that it enabled us to examine the association between sleep and atopic dermatitis activity and severity at multiple time points throughout childhood, allowing a look at sleep outcomes of individuals whose disease was no longer active at any given time point. Children with inactive disease still reported increased odds of impaired sleep quality, at a level similar to those with active but mild disease. Our findings are consistent with previous data from a polysomnographic study: Despite being in a period of clinical remission, children with a history of previously active atopic dermatitis experienced a substantially higher number of arousals and awakenings compared with controls.^28^ Moreover, scratching episodes only accounted for 15% of the arousals and awakenings, suggesting that scratching alone does not explain the sleep fragmentation experienced by these patients.^28^ Fishbein and colleagues^5^ have proposed that this phenomenon may be associated with a heightened sensitivity to sensory stimulation at night secondary to skin damage, which may represent an underlying mechanism of hyperarousability despite good disease control.^6,29^ Other factors that may be implicated in atopic dermatitis–associated sleep disturbances include cytokine and melatonin dysregulation and disrupted circadian rhythms of the skin.^30^

From a clinical perspective, our findings suggest that pediatricians should consider screening all children with atopic dermatitis, even if their disease is mild or no longer active. Clinicians may offer anticipatory guidance, education, behavioral interventions, and referrals if appropriate.^31,32,33^ Early detection and management of sleep problems in children with atopic dermatitis is critical to prevent quality-of-life impairments, daytime fatigue, as well as behavioral and mood disorders reported in children with atopic dermatitis.^2,9,10,34,35^ Additional research is needed to understand whether more aggressive treatment of atopic dermatitis symptoms will lead to improvements in children’s sleep quality.

Sleep disturbances have been studied separately in children with asthma and allergic rhinitis, but few studies have examined differences in sleep quality in children with more than 1 atopic condition.^10,36,37^ One of the strengths of the current study is the inclusion of several time-varying covariates, notably, asthma and allergic rhinitis, which were measured at all of the same time points as the primary exposure. We found that children with both atopic dermatitis and comorbid asthma or allergic rhinitis reported substantially more sleep-quality impairments, which has important clinical and therapeutic implications. This result suggests that children with several atopic diseases may represent a group at higher risk of experiencing disrupted sleep and its consequences, including impaired quality of life, daytime fatigue, poor school performance, and behavioral problems. Clinicians caring for children with several atopic conditions should inquire about nocturnal symptoms and sleep disturbances during routine clinic visits and should consider treating these conditions more aggressively.

### Limitations

This study has several limitations that warrant discussion. As in all large-scale longitudinal studies, the study was missing data and attrition occurred over time. For this reason, we repeated the analyses after conducting multiple imputation and found that the results were similar, which helped address concerns of potential selection bias.

Another important limitation was the possibility for misclassification bias, because both exposure and outcomes were parent- or self-reported. Previous studies have found that parental report closely approximates physician assessment of atopic dermatitis,^38^ and the estimates of the annual period prevalence of atopic dermatitis were consistent with UK estimates from the population-based ISAAC study, which included physical assessment in childhood.^39^ Our findings for both sleep duration and quality were highly consistent with smaller studies that used objective measures of sleep. Comparisons between parental assessment of children’s sleep and objective measures, including polysomnography and actigraphy, have found that parents tend to overestimate sleep duration and underestimate nighttime awakenings,^40,41,42^ both of which would tend to bias our results toward the null. Although our composite measure for sleep quality has not been validated, it included items that are similar to those in the Children’s Sleep Habits Questionnaire, a validated and reliable screening instrument to identify sleep problems in school-aged children.^43^ In addition, although our cohort is fairly representative of the UK population,^14^ the extent to which our results are generalizable to other settings is unclear.

Notwithstanding these limitations, this study has several implications for future research and clinical care. Currently, only a few atopic dermatitis clinical outcome measures address sleep and may not adequately capture the extent of sleep-quality disturbances.^44,45^ Our findings support the development of standardized and validated clinical outcome measures of sleep disturbance that explicitly address several aspects of sleep quality.^45,46^ This refinement would enable future trials to assess the effectiveness of atopic dermatitis interventions in reducing poor sleep.

## Conclusions

Atopic dermatitis appeared to negatively affect sleep quality throughout childhood, even among patients with mild and inactive disease. Increasing disease severity and comorbid asthma or allergic rhinitis appeared to be associated with worse sleep-quality outcomes. Clinical outcome measures for atopic dermatitis should explicitly address sleep quality, and additional work should investigate interventions to improve sleep quality and examine the association between atopic dermatitis treatment and children’s sleep.

## Notes

Supplement. **eTable 1.** Atopic Dermatitis Disease Activity and Severity by Child Age **eTable 2**. Unadjusted Odds of Sleep Disturbances for Children with Active Atopic Dermatitis by Child Age Compared to Children Who Never Reported Atopic Dermatitis **eTable 3.** Subject-specific Odds of Waking at Least Once per Night According to Atopic Dermatitis Disease Activity and Severity Stratified by Comorbid Asthma or Allergic Rhinitis Compared to Children Who Never Reported Atopic Dermatitis **eTable 4.** Subject-specific Odds of Having Difficulty Falling Asleep According to Atopic Dermatitis Disease Activity and Severity Stratified by Comorbid Asthma or Allergic Rhinitis Compared to Children Who Never Reported Atopic Dermatitis **eTable 5.** Subject-specific Odds of Early Morning Awakening According to Atopic Dermatitis Disease Activity and Severity Stratified by Comorbid Asthma or Allergic Rhinitis Compared to Children Who Never Reported Atopic Dermatitis **eTable 6.** Subject-specific Odds of Nightmares According to Atopic Dermatitis Disease Activity and Severity Stratified by Comorbid Asthma or Allergic Rhinitis Compared to Children Who Never Reported Atopic Dermatitis **eTable 7.** Estimated Differences in Nighttime and Total Sleep Duration According to Atopic Dermatitis Disease Activity and Severity Compared to Children Who Never Reported Atopic Dermatitis Using Imputed Data **eTable 8.** Subject-specific Odds of Individual Sleep Quality Disturbances According to Atopic Dermatitis Disease Activity and Severity Compared to Children Who Never Reported Atopic Dermatitis Using Imputed Data
