# Supplementary material for: Association of Atopic Dermatitis With Sleep Quality in Children
Source: JAMA Pediatr. 2019 Mar 4;173(5):e190025. doi: 10.1001/jamapediatrics.2019.0025 (PMC6503516; doi:10.1001/jamapediatrics.2019.0025)
Supplement: Supplement. — eTable 1. Atopic Dermatitis Disease Activity and Severity by Child Age eTable 2. Unadjusted Odds of Sleep Disturbances for Children with Active Atopic Dermatitis by Child Age Compared to Children Who Never Reported Atopic Dermatitis eTable 3. Subject-specific Odds of Waking at Least Once per Night According to Atopic Dermatitis Disease Activity and Severity Stratified by Comorbid Asthma or Allergic Rhinitis Compared to Children Who Never Reported Atopic Dermatitis eTable 4. Subject-specific Odds of Having Difficulty Falling Asleep According to Atopic Dermatitis Disease Activity and Severity Stratified by Comorbid Asthma or Allergic Rhinitis Compared to Children Who Never Reported Atopic Dermatitis eTable 5. Subject-specific Odds of Early Morning Awakening According to Atopic Dermatitis Disease Activity and Severity Stratified by Comorbid Asthma or Allergic Rhinitis Compared to Children Who Never Reported Atopic Dermatitis eTable 6. Subject-specific Odds of Nightmares According to Atopic Dermatitis Disease Activity and Severity Stratified by Comorbid Asthma or Allergic Rhinitis Compared to Children Who Never Reported Atopic Dermatitis eTable 7. Estimated Differences in Nighttime and Total Sleep Duration According to Atopic Dermatitis Disease Activity and Severity Compared to Children Who Never Reported Atopic Dermatitis Using Imputed Data eTable 8. Subject-specific Odds of Individual Sleep Quality Disturbances According to Atopic Dermatitis Disease Activity and Severity Compared to Children Who Never Reported Atopic Dermatitis Using Imputed Data [file jamapediatr-173-e190025-s001.pdf]

Supplementary Online Content

Ramirez FD, Chen S, Langan SM, et al. Association of atopic dermatitis with sleep quality in children. *JAMA Pediatr.* Published online March 4, 2019. doi:10.1001/jamapediatrics.2019.0025

eTable 1. Atopic Dermatitis Disease Activity and Severity by Child Age

eTable 2. Unadjusted Odds of Sleep Disturbances for Children with Active Atopic Dermatitis by Child Age Compared to Children Who Never Reported Atopic Dermatitis

eTable 3. Subject-specific Odds of Waking at Least Once per Night According to Atopic Dermatitis Disease Activity and Severity Stratified by Comorbid Asthma or Allergic Rhinitis Compared to Children Who Never Reported Atopic Dermatitis

eTable 4. Subject-specific Odds of Having Difficulty Falling Asleep According to Atopic Dermatitis Disease Activity and Severity Stratified by Comorbid Asthma or Allergic Rhinitis Compared to Children Who Never Reported Atopic Dermatitis

eTable 5. Subject-specific Odds of Early Morning Awakening According to Atopic Dermatitis Disease Activity and Severity Stratified by Comorbid Asthma or Allergic Rhinitis Compared to Children Who Never Reported Atopic Dermatitis

eTable 6. Subject-specific Odds of Nightmares According to Atopic Dermatitis Disease Activity and Severity Stratified by Comorbid Asthma or Allergic Rhinitis Compared to Children Who Never Reported Atopic Dermatitis

eTable 7. Estimated Differences in Nighttime and Total Sleep Duration According to Atopic Dermatitis Disease Activity and Severity Compared to Children Who Never Reported Atopic Dermatitis Using Imputed Data

eTable 8. Subject-specific Odds of Individual Sleep Quality Disturbances According to Atopic Dermatitis Disease Activity and Severity Compared to Children Who Never Reported Atopic Dermatitis Using Imputed Data

This supplementary material has been provided by the authors to give readers additional information about their work.

© 2019 Ramirez FD et al. *JAMA Pediatr*.

eTable 1. Atopic Dermatitis Disease Activity and Severity by Child Age

Time point	Total respondents **	Never reported AD [a]		Inactive AD [b]		Active AD [c], No problem		Active AD [c], Mild		Active AD [c], Quite bad		Active AD [c], Very bad		Number with data available for both AD and sleep
		Number	%	Number	%	Number	%	Number	%	Number	%	Number	%	
**30 months** (2 y 6 m)	10,196	4,754	46.6	620	6.1	191	1.9	1,040	10.2	602	5.9	170	1.7	10,195
**42 months** (3 y 6 m)	9,972	4,357	43.7	1,155	11.6	182	1.8	1,065	10.7	513	5.1	149	1.5	9,979
**57 months** (4 y 9 m)	9,335	3,823	41.0	1,291	13.8	191	2.1	1,171	12.5	484	5.2	114	1.2	9,324
**69 months** (5 y 9 m)	8,544	3,323	38.9	1,579	18.5	177	2.1	1,039	12.2	376	4.4	87	1.0	8,532
**81 months** (6 y 9 m)	8,418	3,206	38.1	1,835	21.8	161	1.9	968	11.5	311	3.7	81	1.0	8,408
**103 months** (8 y 7 m)	7,893	2,868	36.3	1,824	23.1	173	2.2	928	11.8	299	3.8	72	0.9	-
**128 months** (10 y 8 m)	7,422	2,602	35.1	2,028	27.3	107	1.4	830	11.2	243	3.3	34	0.5	6,717
**140 months** (11 y 8 m)	7,043	2,359	33.5	1,940	27.6	137	2.0	862	12.2	229	3.3	50	0.7	6,608
**166 months** (13 y 10 m)	6,713	2,227	33.2	2,220	33.1	63	0.9	567	8.5	191	2.9	37	0.6	-
**198 months** (16 y 6 m)*	4,813	1,451	30.2	1,637	34.0	61	1.3	354	7.4	213	4.4	65	1.4	3,227

* Child-reported; ** Includes individuals with AD severity data not missing
Atopic dermatitis (AD), defined as ≥ 2 positive responses to the question: *"Has your child had an itchy, dry skin rash in the joints and creases of his body (e.g. behind the knees, elbows, under the arms) in the past year?"* [a] Never AD defined as never reporting a positive response to the question above; [b] Inactive AD defined as a history of ≥ 2 positive responses to the above question, but a current negative response at the specific time point considered; [c] Active AD defined as a history of ≥ 2 positive responses to the above question, including a current positive response at the specific time point considered

© 2019 Ramirez FD et al. *JAMA Pediatr*.

eTable 2. Unadjusted Odds of Sleep Disturbances for Children with Active Atopic Dermatitis by Child Age Compared to Children Who Never Reported Atopic Dermatitis

**Child age**	**Regularly waking at least once in the night**	**Regularly waking early in the morning**	**Regularly having difficulty falling asleep**	**Regularly experiencing nightmares**	**Composite outcome *: Experiencing more sleep quality disturbances**
	Odds Ratio (95% CI)	Odds Ratio (95% CI)	Odds Ratio (95% CI)	Odds Ratio (95% CI)	Odds Ratio (95% CI)
**30 months**	1.19 (1.04, 1.35)	1.26 (1.11, 1.44)	1.08 (0.94, 1.23)	1.15 (0.99, 1.34)	1.22 (1.09, 1.37)
**42 months**	1.14 (0.99, 1.31)	1.29 (1.12, 1.48)	1.28 (1.12, 1.47)	1.38 (1.20, 1.59)	1.40 (1.24, 1.58)
**57 months**	1.38 (1.19, 1.61)	1.32 (1.14, 1.52)	1.29 (1.12, 1.50)	1.28 (1.11, 1.49)	1.47 (1.29, 1.66)
**69 months**	1.36 (1.14, 1.63)	1.12 (0.95, 1.31)	1.40 (1.19, 1.64)	1.39 (1.19, 1.63)	1.45 (1.27, 1.67)
**81 months**	1.50 (1.23, 1.83)	1.09 (0.92, 1.28)	1.46 (1.23, 1.74)	1.42 (1.20, 1.68)	1.51 (1.30, 1.74)
**115 months**	1.23 (0.89, 1.69)	1.18 (0.93, 1.50)	1.32 (1.04, 1.67)	1.59 (1.23, 2.05)	1.49 (1.21, 1.84)

Results from unadjusted logistic and ordered logistic (*composite outcome measure of sleep quality that combines nighttime awakenings, early morning awakenings, difficulty going to sleep, and nightmares) regression models examining the association between active atopic dermatitis and different sleep quality outcomes at 6 time points between ages 2 and 10.

© 2019 Ramirez FD et al. *JAMA Pediatr*.

eTable 3. Subject-specific Odds of Waking at Least Once per Night According to Atopic Dermatitis Disease Activity and Severity Stratified by Comorbid Asthma or Allergic Rhinitis Compared to Children Who Never Reported Atopic Dermatitis

**Atopic dermatitis disease activity and severity**	**Unadjusted[1]**	**Adjusted[2]**	
		**No asthma or allergic rhinitis**	**Asthma or allergic rhinitis**
	Odds Ratio (95% CI)	Odds Ratio (95% CI)	Odds Ratio (95% CI)
Never reported atopic dermatitis	Ref.	Ref.	Ref.
Overall active atopic dermatitis	0.96 (0.85, 1.08)	1.27 (1.08, 1.49)	1.53 (1.23, 1.90)
No problem	0.65 (0.53, 0.81)	1.02 (0.78, 1.32)	1.43 (0.85, 2.39)
Mild	0.62 (0.55, 0.70)	1.01 (0.88, 1.17)	0.96 (0.76, 1.21)
Quite bad	1.14 (0.99, 1.32)	1.40 (1.16, 1.70)	1.55 (1.17, 2.06)
Very bad	1.83 (1.42, 2.36)	1.80 (1.21, 2.69)	2.55 (1.60, 4.06)
Inactive atopic dermatitis	0.42 (0.38, 0.46)	1.06 (0.92, 1.22)	1.20 (0.96, 1.51)

Overall test for interaction: p = 0.228
Results from unadjusted and adjusted multivariable mixed effects logistic regression models examining the association between atopic dermatitis and nighttime awakening (≥ 1 awakening per night) at 6 time points between ages 2 and 10. The multivariable model adjusted for potential confounders including child gender, child age, child race/ethnicity, comorbid asthma or allergic rhinitis, household smoking exposure, maternal education, social class, crowding index, financial difficulties score, and maternal age at delivery, and included an interaction term between atopic dermatitis and comorbid asthma or allergic rhinitis. Although the test for interaction was not statistically significant, the interaction term was included on theoretical grounds, as it was statistically significant and included in the primary analysis with the composite sleep quality outcome.

[1] Model N = 11,446 individuals [mean of 4.5 observations per individual (range 1-6)]

[2] Model N = 9,089 individuals [mean of 4.5 observations per individual (range 1-6)]

© 2019 Ramirez FD et al. *JAMA Pediatr*.

eTable 4. Subject-specific Odds of Having Difficulty Falling Asleep According to Atopic Dermatitis Disease Activity and Severity Stratified by Comorbid Asthma or Allergic Rhinitis Compared to Children Who Never Reported Atopic Dermatitis

**Atopic dermatitis disease activity and severity**	**Unadjusted[1]**	**Adjusted[2]**	
		**No asthma or allergic rhinitis**	**Asthma or allergic rhinitis**
	Odds Ratio (95% CI)	Odds Ratio (95% CI)	Odds Ratio (95% CI)
Never reported atopic dermatitis	Ref.	Ref.	Ref.
Overall active atopic dermatitis	1.41 (1.28, 1.56)	1.37 (1.20, 1.56)	1.49 (1.24, 1.79)
No problem	1.27 (1.07, 1.52)	1.15 (0.93, 1.43)	1.37 (0.88, 2.12)
Mild	1.47 (1.34, 1.62)	1.34 (1.20, 1.50)	1.49 (1.24, 1.79)
Quite bad	1.41 (1.24, 1.60)	1.32 (1.12, 1.55)	1.66 (1.31, 2.12)
Very bad	1.50 (1.19, 1.88)	1.73 (1.22, 2.45)	1.44 (0.96, 2.16)
Inactive atopic dermatitis	1.85 (1.70, 2.03)	1.33 (1.19, 1.48)	1.51 (1.26, 1.81)

Overall test for interaction: p = 0.005
Results from unadjusted and adjusted multivariable mixed effects logistic regression models examining the association between atopic dermatitis and difficulty falling asleep at 6 time points between ages 2 and 10. The multivariable model adjusted for potential confounders including child gender, child age, child race/ethnicity, comorbid asthma or allergic rhinitis, household smoking exposure, maternal education, social class, crowding index, financial difficulties score, and maternal age at delivery, and included an interaction term between atopic dermatitis and comorbid asthma or allergic rhinitis.
[1] Model N = 11,459 individuals [mean of 4.6 observations per individual (range 1-6)]
[2] Model N = 9,102 individuals [mean of 4.5 observations per individual (range 1-6)]

© 2019 Ramirez FD et al. *JAMA Pediatr*.

eTable 5. Subject-specific Odds of Early Morning Awakening According to Atopic Dermatitis Disease Activity and Severity Stratified by Comorbid Asthma or Allergic Rhinitis Compared to Children Who Never Reported Atopic Dermatitis

**Atopic dermatitis disease activity and severity**	**Unadjusted[1]**	**Adjusted[2]**	
		**No asthma or allergic rhinitis**	**Asthma or allergic rhinitis**
	Odds Ratio (95% CI)	Odds Ratio (95% CI)	Odds Ratio (95% CI)
Never reported atopic dermatitis	Ref.	Ref.	Ref.
Overall active atopic dermatitis	1.15 (1.03, 1.28)	1.40 (1.21, 1.61)	1.56 (1.27, 1.91)
No problem	0.98 (0.81, 1.19)	1.22 (0.97, 1.54)	1.48 (0.92, 2.38)
Mild	1.03 (0.93, 1.14)	1.37 (1.21, 1.55)	1.39 (1.14, 1.70)
Quite bad	1.22 (1.06, 1.40)	1.25 (1.05, 1.49)	1.84 (1.41, 2.40)
Very bad	1.41 (1.09, 1.82)	1.81 (1.24, 2.66)	1.57 (1.00, 2.45)
Inactive atopic dermatitis	0.82 (0.75, 0.91)	1.34 (1.19, 1.51)	1.36 (1.12, 1.66)

Overall test for interaction: p = 0.078
Results from unadjusted and adjusted multivariable mixed effects logistic regression models examining the association between atopic dermatitis and early morning awakening at 6 time points between ages 2 and 10. The multivariable model adjusted for potential confounders including child gender, child age, child race/ethnicity, comorbid asthma or allergic rhinitis, household smoking exposure, maternal education, social class, crowding index, financial difficulties score, and maternal age at delivery, and included an interaction term between atopic dermatitis and comorbid asthma or allergic rhinitis. Although the test for interaction was not statistically significant, the interaction term was included on theoretical grounds, as it was statistically significant and included in the primary analysis with the composite sleep quality outcome.
[1] Model N = 11,459 individuals [mean of 4.6 observations per individual (range 1-6)]
[2] Model N = 9,102 individuals [mean of 4.5 observations per individual (range 1-6)]

© 2019 Ramirez FD et al. *JAMA Pediatr*.

eTable 6. Subject-specific Odds of Nightmares According to Atopic Dermatitis Disease Activity and Severity Stratified by Comorbid Asthma or Allergic Rhinitis Compared to Children Who Never Reported Atopic Dermatitis

Atopic dermatitis disease activity and severity	Unadjusted[1]	Adjusted[2]	
		No asthma or allergic rhinitis	Asthma or allergic rhinitis
	Odds Ratio (95% CI)	Odds Ratio (95% CI)	Odds Ratio (95% CI)
Never reported atopic dermatitis	Ref.	Ref.	Ref.
Overall active atopic dermatitis	1.56 (1.40, 1.74)	1.47 (1.28, 1.69)	1.71 (1.41, 2.07)
No problem	1.35 (1.12, 1.63)	1.28 (1.02, 1.60)	1.40 (0.89, 2.22)
Mild	1.59 (1.44, 1.76)	1.45 (1.29, 1.64)	1.56 (1.28, 1.89)
Quite bad	1.65 (1.44, 1.89)	1.53 (1.29, 1.81)	1.80 (1.40, 2.30)
Very bad	1.67 (1.31, 2.12)	1.63 (1.14, 2.34)	2.19 (1.45, 3.31)
Inactive atopic dermatitis	1.68 (1.53, 1.84)	1.46 (1.31, 1.63)	1.46 (1.22, 1.75)

Overall test for interaction: p = 0.873
Results from unadjusted and adjusted multivariable mixed effects logistic regression models examining the association between atopic dermatitis and nightmares at 6 time points between ages 2 and 10. The multivariable model adjusted for potential confounders including child gender, child age, child race/ethnicity, comorbid asthma or allergic rhinitis, household smoking exposure, maternal education, social class, crowding index, financial difficulties score, and maternal age at delivery, and included an interaction term between atopic dermatitis and comorbid asthma or allergic rhinitis. Although the test for interaction was not statistically significant, the interaction term was included on theoretical grounds, as it was statistically significant and included in the primary analysis with the composite sleep quality outcome.
[1] Model N = 11,457 individuals [mean of 4.6 observations per individual (range 1-6)]
[2] Model N = 9,100 individuals [mean of 4.5 observations per individual (range 1-6)]

© 2019 Ramirez FD et al. *JAMA Pediatr*.

eTable 7. Estimated Differences in Nighttime and Total Sleep Duration According to Atopic Dermatitis Disease Activity and Severity Compared to Children Who Never Reported Atopic Dermatitis Using Imputed Data

**Nighttime duration**	**Unadjusted**	**Adjusted**
**Atopic dermatitis disease activity and severity**	**Estimated difference in nighttime sleep duration in minutes (95% CI)**	**Estimated difference in nighttime sleep duration in minutes (95% CI)**
Never reported atopic dermatitis	Ref.	Ref.
Active atopic dermatitis		
No problem	-14 (-18, -9)	1 (-1, 4)
Mild	-13 (-15, -10)	1 (0, 3)
Quite bad	-12 (-16, -9)	1 (-1, 3)
Very bad	-14 (-21, -7)	2 (-3, 6)
Inactive atopic dermatitis	-41 (-43, -39)	3 (1, 4)
**Total duration**	**Unadjusted**	**Adjusted**
**Atopic dermatitis disease activity and severity**	**Estimated difference in total sleep duration in minutes (95% CI)**	**Estimated difference in total sleep duration in minutes (95% CI)**
Never reported atopic dermatitis	Ref.	Ref.
Active atopic dermatitis		
No problem	-15 (-20, -11)	-2 (-5, 1)
Mild	-15 (-17, -12)	-2 (-3, 0)
Quite bad	-12 (-15, -8)	-1 (-3, 1)
Very bad	-12 (-19, -4)	0 (-4, 4)
Inactive atopic dermatitis	-47 (-49, -45)	-1 (-2, 1)

Results using imputed data, from unadjusted and adjusted multivariable generalized estimating equation (GEE) regression models examining the association between atopic dermatitis and nighttime and total sleep duration at 8 time points between ages 2 and 16. The multivariable model adjusted for potential confounders including child gender, child age, child race/ethnicity, comorbid asthma or allergic rhinitis, household smoking exposure, maternal education, social class, crowding index, financial difficulties score, and maternal age at delivery. Due to the extremely large size of the imputed dataset, mixed models were not computationally feasible and GEE models were used instead. Robust standard errors were used in the GEE models.

© 2019 Ramirez FD et al. *JAMA Pediatr*.

eTable 8. Subject-specific Odds of Individual Sleep Quality Disturbances According to Atopic Dermatitis Disease Activity and Severity Compared to Children Who Never Reported Atopic Dermatitis Using Imputed Data

**Waking at least once per night**	**Unadjusted**	**Adjusted (AD alone)**
**Atopic dermatitis disease activity and severity**	**Odds Ratio (95% CI)**	**Odds Ratio (95% CI)**
Never reported atopic dermatitis	Ref.	Ref.
Active atopic dermatitis		
No problem	0.92 (0.79, 1.06)	1.05 (0.92, 1.20)
Mild	0.90 (0.83, 0.98)	1.06 (0.97, 1.15)
Quite bad	1.25 (1.14, 1.38)	1.26 (1.13, 1.41)
Very bad	1.74 (1.49, 2.03)	1.38 (1.13, 1.68)
Inactive atopic dermatitis	0.78 (0.73, 0.83)	1.01 (0.93, 1.09)
**Difficulty falling asleep**	**Unadjusted**	**Adjusted (AD alone)**
**Atopic dermatitis disease activity and severity**	**Odds Ratio (95% CI)**	**Odds Ratio (95% CI)**
Never reported atopic dermatitis	Ref.	Ref.
Active atopic dermatitis		
No problem	1.25 (1.11, 1.42)	1.14 (1.00, 1.30)
Mild	1.36 (1.27, 1.25)	1.21 (1.12, 1.30)
Quite bad	1.32 (1.21, 1.44)	1.21 (1.09, 1.34)
Very bad	1.35 (1.16, 1.57)	1.34 (1.10, 1.63)
Inactive atopic dermatitis	1.56 (1.47, 1.66)	1.23 (1.15, 1.31)

© 2019 Ramirez FD et al. *JAMA Pediatr*.

eTable8. (continued)

Early morning awakening	Unadjusted	Adjusted (AD alone)
**Atopic dermatitis disease activity and severity**	**Odds Ratio (95% CI)**	**Odds Ratio (95% CI)**
Never reported atopic dermatitis	Ref.	Ref.
Active atopic dermatitis		
No problem	1.01 (1.00, 1.13)	1.18 (1.03, 1.35)
Mild	1.02 (1.00, 1.10)	1.22 (1.13, 1.32)
Quite bad	1.13 (1.03, 1.24)	1.22 (1.10, 1.35)
Very bad	1.25 (1.08, 1.46)	1.31 (1.08, 1.58)
Inactive atopic dermatitis	0.88 (0.82, 0.93)	1.20 (1.12, 1.29)
**Nightmares**	**Unadjusted**	**Adjusted (AD alone)**
**Atopic dermatitis disease activity and severity**	**Odds Ratio (95% CI)**	**Odds Ratio (95% CI)**
Never reported atopic dermatitis	Ref.	Ref.
Active atopic dermatitis		
No problem	1.27 (1.12, 1.45)	1.24 (1.08, 1.42)
Mild	1.41 (1.32, 1.52)	1.36 (1.26, 1.47)
Quite bad	1.45 (1.32, 1.59)	1.38 (1.24, 1.53)
Very bad	1.52 (1.30, 1.78)	1.34 (1.10, 1.62)
Inactive atopic dermatitis	1.39 (1.30, 1.49)	1.32 (1.23, 1.42)

Results using imputed data, from unadjusted and adjusted multivariable generalized estimating equation (GEE) regression models examining the association between atopic dermatitis and four individual sleep quality disturbances at 6 time points between ages 2 and 10. The multivariable model adjusted for potential confounders including child gender, child age, child race/ethnicity, comorbid asthma or allergic rhinitis, household smoking exposure, maternal education, social class, crowding index, financial difficulties score, and maternal age at delivery, and included an interaction term between atopic dermatitis and comorbid asthma or allergic rhinitis. Due to the extremely large size of the imputed dataset, mixed models were not computationally feasible and GEE models were used instead. Robust standard errors were used in the GEE models.

© 2019 Ramirez FD et al. *JAMA Pediatr*.
